# The Role of Physical Stabilization in Whole Blood Preservation

**DOI:** 10.1038/srep21023

**Published:** 2016-02-15

**Authors:** Keith H. K. Wong, Rebecca D. Sandlin, Thomas R. Carey, Kathleen L. Miller, Aaron T. Shank, Rahmi Oklu, Shyamala Maheswaran, Daniel A. Haber, Daniel Irimia, Shannon L. Stott, Mehmet Toner

**Affiliations:** 1 BioMEMS Resource Center, Center for Engineering in Medicine, & Department of Surgery, Massachusetts General Hospital, Harvard Medical School, Boston, MA 02114, USA; 2 Department of Radiology, Massachusetts General Hospital, Harvard Medical School, Boston, MA 02114, USA; 3 Mayo Clinic, Division of Interventional Radiology, Scottsdale, AZ 85259, USA; 4 Cancer Center & Department of Surgery, Massachusetts General Hospital, Harvard Medical School, Boston, MA 02114, USA; 5 Cancer Center & Department of Medicine, Massachusetts, MA General Hospital, Harvard Medical School, Boston, MA 02114, USA; 6 Howard Hughes Medical Institute, Chevy Chase, MD 20815, USA; 7 Cancer Center, Department of Medicine, & BioMEMS Resource Center, Center for Engineering in Medicine, Massachusetts General Hospital, Harvard Medical School, Boston, MA 02114, USA.

## Abstract

The rapid degradation of blood *ex vivo* imposes logistical limitations on the utilization of blood-borne cells in medical diagnostics and scientific investigations. A fundamental but overlooked aspect in the storage of this fluid tissue is blood settling, which induces physical stress and compaction, aggregates blood cells, and causes collateral damage due to leukocyte activation. Here we show that the polymer Ficoll 70 kDa stabilized blood samples and prevented blood settling over the course of 72 hours, primarily by inhibiting depletion-mediated red blood cell aggregation. Physical stabilization decreased echinocyte formation, improved leukocyte viability, and inhibited the release of neutrophil elastase—a marker of neutrophil extracellular trap formation. In addition, Ficoll-stabilized blood was compatible with common leukocyte enrichment techniques including red blood cell lysis and immunomagnetic purification. This study showed for the first time that blood settling can be prevented using polymers and has implications in diagnostics.

Peripheral blood is the most frequently accessed tissue in the clinic, and the isolation of blood-borne cells is of broad clinical and scientific importance in hematology, transfusion, immunology, regenerative medicine, and oncology^1^,^2^,^3^,^4^. More recently, developments in microfluidics and lab-on-a-chip devices have greatly advanced our capabilities in isolating pure populations of cells and performing high-throughput, multidimensional assays^5^. The rapidly growing field of microfluidic technologies has expanded into applications ranging from T cell isolation for HIV disease monitoring^6^ and multiplexed detection of cytokine secretion^7^; gene expression profiling of neutrophils in trauma and burn patients^8^; enrichment of CD34 + hematopoietic stem cells^9^; to minimally invasive detection of nucleated red blood cells (RBCs) from maternal blood^10^ as well as rare circulating tumor cells for cancer diagnosis^11^ and identification of druggable mutations^12^.

Similar to any tissue, whole blood (WB) deteriorates quickly *ex vivo*. In modern transfusion medicine, WB is typically processed into various components for specialized storage within 24 hours^3^. The short lifespan of neutrophils further limits the use of WB in transfusion^13^. Given that many clinically relevant assays such as sequencing and expression profiling are best performed in large medical centers or diagnostic laboratories, the logistical needs of blood storage and transportation impose severe limitations on the dissemination of next-generation blood-based medical technologies.

The current paradigm in blood storage—anti-coagulation, pH buffering, and energy supplementation—has remained unchanged for decades. However, a fundamental aspect in the storage of this fluid tissue, blood cell settling, has been largely overlooked. This physical event alters cellular activities and leads to platelet aggregation^14^,^15^ and functional deterioration of leukocytes^16^,^17^. Damaged cells are physically compacted in a confined space and liberate toxic byproducts that cross-activate each other, causing collateral damage to otherwise healthy cells. Although sample mixing during storage retards cellular degradation^18^, this approach is not feasible during sample shipment and may cause damage due to mechanical stresses, for instance hemolysis and platelet activation^16^,^19^,^20^. The ability to suspend blood in a homogeneous phase may therefore improve the preservation of WB *ex vivo* and facilitate the logistics of off-site or delayed processing of samples.

In this work, we introduce a method to prevent the sedimentation of WB in an attempt to improve the viable preservation of blood samples *ex vivo*. To stabilize materials in the fluid phase, we borrowed ideas from industrial products (e.g., shampoo and ketchup) that contain polymer additives (e.g., xanthan gum, corn starch) as thickeners. These polymers prevent phase separation of the liquid components primarily through modifying rheological properties such as increasing the low-shear viscosity. A similar strategy has been employed in the popular methylcellulose assay, in which the semi-solid culture medium separates hematopoietic stem cells for the study of colony formation. To achieve suspended storage of blood, we chose the polysaccharide Ficoll, which is highly biocompatible due to its neutral charge and high hydrophilicity. We first tested the ability of Ficoll polymers in preventing blood settling over the course of 3 days in ambient temperature and explored the associated mechanisms. We then studied whether this treatment affects routine blood cell enrichment methodologies, and the effects of physical stabilization on the morphology, viability, and various biological processes of blood cells in storage.

## Results

### Biophysical stabilization of blood

**Figure 1:**
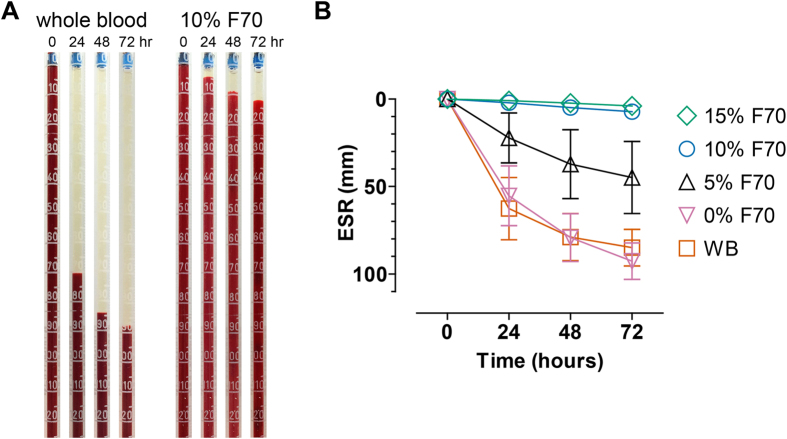
(**A**) Representative images that show the measurement of ESR on WB (left) and blood that contains 10% F70 (right). Note that these images do not represent the entire length of the ESR assay. (**B**) ESR of blood samples in WB and blood containing different concentrations of F70 over 72 hours (WB, *n* = 11; 0% F70, *n* = 9; 5% F70, *n* = 8; 10% F70, *n* = 11; 15% F70, *n* = 9). 0% F70 represents the control condition which was treated with RPMI medium without F70. Error bars represent the 95% confidence interval (CI) of the mean.

We quantified the erythrocyte settling rate (ESR) with standardized pipets used in the Westergren method (Fig. 1A). Whole blood in the absence of Ficoll settled quickly, reaching 62.6 ± 26.5 mm at 24 hours and further to 78.9 ± 20.0 and 84.9 ± 15.6 mm at 48 and 72 hours respectively (Fig. 1B). To introduce Ficoll 70 kDa (F70) into blood, we mixed concentrated stock solutions of F70 (dissolved in RPMI media) to blood samples while maintaining the dilution ratio constant at 75% blood volume fraction. The addition of 5%, 10%, and 15% F70 greatly decreased the ESR at all measured timepoints (Fig. 1B; *p* < 0.0001 compared to WB at 24, 48, and 72 hours). Most impressively, 10% and 15% F70 almost completely prevented settling over the course of 72 hours (ESRs were 7.3 ± 3.1 mm and 4 ± 1.6 mm, respectively; Fig. 1B). To confirm that the stabilization effects were due to F70 but not a result of dilution with media, we added RPMI to WB at the same ratio and found no difference in ESR (0% F70, Fig. 1B; *p* > 0.05 compared to WB at all timepoints).

**Figure 2:**
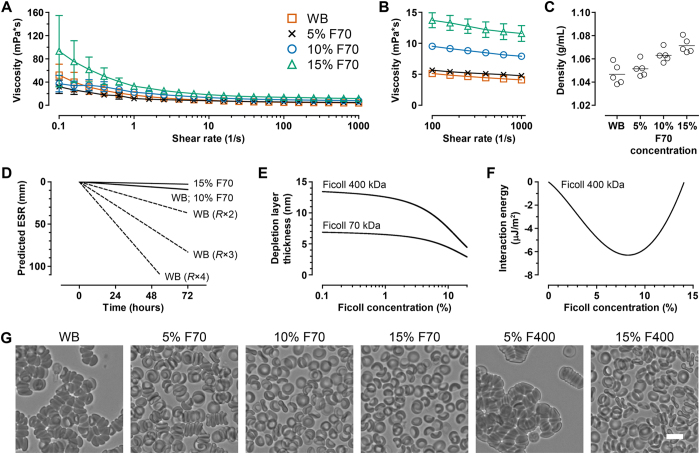
(**A**) Viscosities of blood samples as a function of shear rate (WB, *n* = 5; 5% F70, *n* = 4; 10% F70, *n* = 5; 15% F70, *n* = 5). (**B**) Viscosities of blood samples in (**A**) focusing on the high-shear range for easier visualization. (**C**) Densities of blood samples containing different concentrations of F70. (**D**) Estimated ESR values calculated from the settling velocity. Settling as single cells is extremely slow regardless of the medium (WB, 10% F70, and 15% F70). However, cellular aggregation effectively increases the radius of the particle (i.e., *R* × 2, *R* × 3, etc.) and thereby greatly increases the rate of settling. (**E**) The computed thickness of the depletion layer on the surface of RBCs in the presence of Ficoll 70 kDa or 400 kDa polymers at a range of bulk concentrations. (**F**) The minimum interaction energy of Ficoll 400 kDa in a range of bulk concentrations. Negative energies are indicative of attractive forces which cause RBC aggregation. Interaction energies due to Ficoll 70 kDa are universally non-negative and are therefore not shown. (**G**) When samples were left undisturbed for 20 minutes, RBCs in WB formed aggregates spontaneously. RBC aggregation was greatly inhibited in the presence of 5% F70, and completely prevented in the presence of 10% and 15% F70. In contrast, RBCs aggregated immediately in the presence of 5% F400, but 15% F400 also inhibited aggregation. Scale bar represents 10 μm.

To understand how F70 stabilized blood, we characterized the rheological properties that determine the settling rate. If we examine a simple scenario of a single sphere settling in a viscous fluid, the settling velocity *v*_*s*_ can be calculated by considering the weight of the sphere, buoyancy force, and the Stokes’ drag force, leading to the classical result


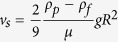


where *ρ*_*p*_ is the sphere density, *ρ*_*f*_ is the fluid density, *μ* is the fluid viscosity, *g* is gravity, and *R* is the radius of the sphere. We first characterized the viscosity profiles of WB and Ficoll-blood samples using Couette viscometry. Whole blood displayed shear-thinning behavior, with viscosity decreasing from 51.9 ± 18.9 mPa·s at a shear rate of 0.1/s to 4.1 ± 0.4 mPa·s at a shear rate of 1000/s (Fig. 2A). The addition of 15% F70 increased the low-shear viscosity of blood samples (*p* < 0.01 at shear rates ≤ 0.398/s, compared to WB) and retained a shear-thinning profile (Fig. 2A). Addition of 5% and 10% F70 did not lead to significant alterations in viscosity compared to WB, but interestingly their low-shear viscosities were lower than 15% F70 (Fig. 2A; *p* < 0.05 at shear rates ≤ 0.631/s for 5% vs. 15% F70; *p* < 0.01 at shear rates ≤ 0.251/s for 10% vs. 15% F70). The densities of WB, 5% F70, 10% F70, and 15% F70 were 1.047 ± 0.009 g/mL, 1.051 ± 0.007 g/mL, 1.063 ± 0.006 g/mL, and 1.071 ± 0.006 g/mL, respectively (Fig. 2C; *p* < 0.0001, 1-way ANOVA with post-test for linear trend).

The minimal increases in densities (2.3% only for 15% F70) and insignificant changes in viscosities (for 5% and 10% F70 vs. WB) appeared insufficient to explain how Ficoll stabilized blood. In fact, using the settling velocity as a first approximation, the ESR is estimated to be minimal (<10 mm at 72 hours) even in WB so long as RBCs settle as single cells (Fig. 2D). However, settling rate would greatly increase in the event of RBC aggregation because *v*_*s*_ scales with *R*^2^ (Fig. 2D). We experimentally confirmed that RBCs in WB aggregated quickly within minutes, whereas the addition of 5% F70 greatly inhibited aggregation (Fig. 2G). Notably, 10% and 15% F70 completely prevented aggregation (Fig. 2G) and sufficiently explained the agreement between the estimated and measured ESR values in these two experimental conditions.

### Red blood cell preservation

**Figure 3:**
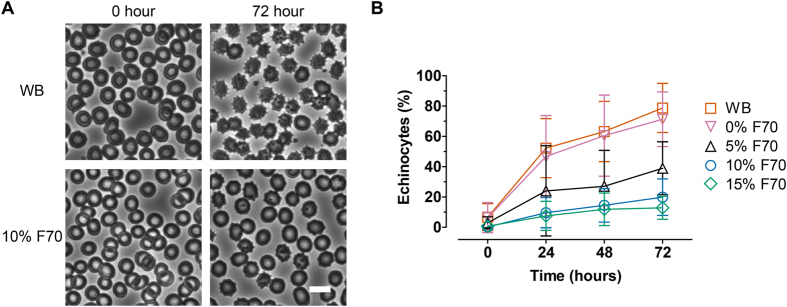
(**A**) Representative phase-contrast images of RBCs in WB or 10% F70 stored for 0 or 72 hours. Echinocytes are RBCs that contain spiculations. Scale bar represents 10 μm. (**B**) Percentages of echinocytes in blood samples stored in different F70 concentrations over 72 hours (WB, *n* = 9; 0% F70, *n* = 8; 5% F70, *n* = 6; 10% F70, *n* = 7; 15% F70, *n* = 6). Error bars represent the 95% confidence interval (CI) of the mean.

We proceeded to determine whether physical stabilization of blood improved the preservation of RBCs. A distinctive feature of RBC aging is their conversion into echinocytes which are characterized by the loss of biconcave disc morphology and emergence of spiculations (Fig. 3A). We quantified the percentage of echinocytes as a result of storage in settled WB or Ficoll-stabilized blood (Fig. 3B). Echinocyte levels in WB increased from 6.6 ± 11.6% at 0 hour to 52.2 ± 25.4%, 63.2 ± 25.9%, and 78.8 ± 21.1% at 24, 48, and 72 hours, respectively. Addition of 5%, 10%, or 15% F70 significantly decreased echinocyte levels post-storage (Fig. 3B; *p* < 0.05, <0.01, <0.001 for 5% F70 vs. WB at 24, 48, & 72 hours, respectively; *p* < 0.001 for 10% or 15% F70 vs. WB at 24, 48, & 72 hours). Dilution with RPMI (0% F70) had no effect on echinocyte formation as expected (*p* > 0.05 at all timepoints).

In some applications (e.g., transfusion) it may be desirable to wash and resuspend blood cells in media or buffer instead of plasma. It is therefore of interest whether the presence of F70 is necessary to maintain RBC morphology. We found that the protective effect of F70 in preventing echinocyte formation post-storage was still present after extensive washing with PBS (Fig. S1). Together, these results demonstrate that Ficoll improves the preservation of RBCs.

### Compatibility with leukocyte enrichment methodologies

Many assays require enrichment steps that isolate the desired cell populations in high purity. We determined whether F70-stabilized blood is compatible with common techniques for leukocyte enrichment. We found that F70 did not affect routine hypotonic lysis of RBCs and resulted in leukocyte yields comparable to WB samples (46.7 ± 1.8% for WB vs. 43.9 ± 1.9% for 10% F70, *n* = 3 each, *p* = 0.2). Flow cytometric analysis indicated no alterations in the expression of the leukocyte marker, CD45 (relative fluorescence, 54 ± 13 × 10^3^ for WB vs. 56 ± 21 × 10^3^ for 10% F70, *n* = 6 each, *p* = 1.0), suggesting compatibility with common surface antigen-based enrichment techniques. We further tested an immunomagnetic negative selection assay for neutrophil enrichment and obtained equivalent yield (30.7% ± 8.2% for WB vs. 29.2% ± 10.5% for 10% F70, *n* = 4 each, *p* = 0.89) and purity (99.0% ± 1.0% for WB vs. 99.0% ± 0.8% for 10% F70, *n* = 4 each, *p* = 1.0). Importantly, treatment with Ficoll did not activate neutrophils (CD11b relative fluorescence, 123 ± 36 × 10^3^ for WB vs. 123 ± 15 × 10^3^ for 10% F70, *n* = 4 each, *p* = 0.89). These results suggest that stabilization of blood by Ficoll does not adversely affect the physiology and surface marker expression of blood cells.

### Leukocyte preservation

**Figure 4:**
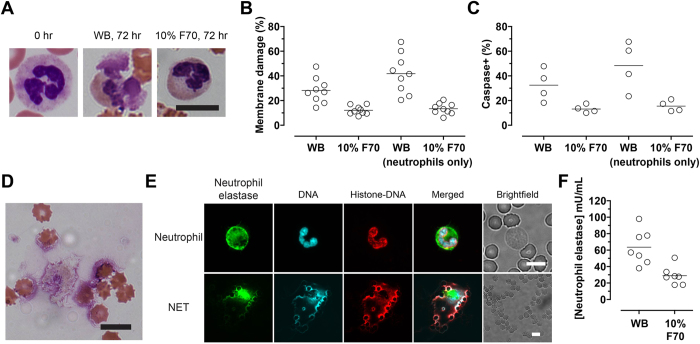
(**A**) High-power (100×) microscopic images of stained neutrophils in fresh blood (0 hr) and blood stored for 72 hours either as WB or in 10% F70. The nuclei of fresh neutrophils display the distinct segmented, multilobular morphology (0 hr), which is better preserved in 10% F70 than WB. Scale bar represents 10 μm. (**B**) Percentages of leukocytes or neutrophils that stained positive for Sytox Blue (i.e., membrane-compromised) after storage in WB or 10% F70 for 72 hours. (**C**) Percentages of leukocytes or neutrophils that stained positive for caspase-3/7 activity after storage in WB or 10% F70 for 72 hours. (**D**) Highly dispersed nuclear materials accompanying cytoplasmic remnants were found in stored blood. Scale bar represents 10 μm. (**E**) Immunofluorescence images of a healthy neutrophil (top row) and a neutrophil extracellular trap (NET; bottom row) stained with antibodies to neutrophil elastase and the histone-DNA complex, and DAPI. Note the large area of NET compared to single RBCs in the brightfield image. Scale bars represent 10 μm. (**F**) Quantification of neutrophil elastase in blood samples stored for 72 hours. In fresh blood samples, neutrophil elastase was detected at a level of 37.8 ± 16.5 mU/mL of blood (*n* = 7). As a positive control, fresh WB stimulated with phorbol myristate acetate (a potent inducer of NETs; *n* = 7) increased the level to 81.4 ± 64.4 mU/mL.

To test whether physical stabilization improved the preservation of leukocytes, we observed the morphology of leukocytes on Wright-Giemsa-stained blood smears and assayed cell viability using flow cytometry. Neutrophils and their distinct multilobular nuclear morphology showed clear signs of disintegration after storage in WB for 72 hours. In comparison, neutrophil morphology was better preserved in Ficoll-stabilized blood (Fig. 4A). To study events related to cell death, we performed imaging flow cytometry using stains that identify membrane-compromised (Sytox Blue) as well as apoptotic (positive for caspase-3/7 activity) cells. After 72 hours of storage in WB, 28.2 ± 10.2% of all leukocytes and 41.8 ± 15.9% of neutrophils stained positive for Sytox (Fig. 4B). In contrast, blood stabilized in 10% F70 displayed significantly higher viability (12.1 ± 3.5% Sytox-positive in all leukocytes, *p* = 0.0009; 13.4 ± 4.5% Sytox-positive in neutrophils, *p* < 0.0001; Fig. 4B). Results for caspase-3/7 activity followed a similar trend. In WB, 32.5 ± 13.1% of all leukocytes and 48.3% ± 19.9% of neutrophils were caspase-positive post-storage, compared to 13.2 ± 3.3% for all leukocytes (*p* = 0.0286) and 15.6 ± 4.5% (*p* = 0.0286) for neutrophils in Ficoll-stabilized blood (Fig. 4C). Overall, more than 93% of cells that stained positive for Sytox post-storage also stained positive for caspase activity, suggesting that the apoptotic program was activated in the majority of non-viable cells. These results demonstrate that Ficoll prevents cell death in blood storage, and is remarkably effective in inhibiting the degradation of neutrophils.

Recently, neutrophil extracellular traps (NETs) have been found in stored RBC units that were not leukoreduced^21^. The evident degradation of neutrophils led us to investigate NET formation. On blood smears obtained from stored samples, we observed highly dispersed nuclear materials that often spanned a large area (Fig. 4D), suggesting the formation of NETs. Immunostaining of the neutrophil-specific marker neutrophil elastase as well as the histone-DNA complex confirmed the presence of NETs in stored blood samples (Fig. 4E)^22^. To quantify the extent of NETosis, we measured the plasma levels of neutrophil elastase post-storage with ELISA. We found a 2.2-fold difference in elastase in WB compared to 10% F70 at 72 hours (63.6 ± 21.1 mU/mL vs. 29 ± 11.2 mU/mL; *p* = 0.0023; Fig. 4F). These results suggest that Ficoll is effective in inhibiting NETosis.

## Discussion

Improving the preservation of peripheral blood opens up opportunities for a wide range of clinical and scientific applications. Preservation in the viable state is important not only for applications such as transfusion and tissue regeneration, but also for diagnostic tests that require high-quality molecular materials which can be severely compromised during fixation^23^. Despite developments in preservation solutions that are designed for purified blood components such as platelets and RBCs, progress in the preservation of WB has been relatively limited, and no attempts have been made to address the fundamental issue of blood settling. Here we show that blood settling and the associated cellular degradation can be minimized by physical stabilization. Introduction of Ficoll polymers into blood is simple and is compatible with common assays for leukocyte enrichment. Physical stabilization inhibited red blood cell aggregation and echinocyte formation, maintained leukocyte viability, and prevented NETosis of neutrophils.

Blood settling in WB is mainly driven by spontaneous RBC aggregation in the presence of large plasma proteins, of which fibrinogen (~340 kDa; hydrodynamic radius ~11 nm) is the most extensively studied^24^,^25^. The molecular forces that drive RBC aggregation can be described by the depletion interaction mechanism^26^, which states that large macromolecules (radii >>4 nm) are preferentially excluded near the RBC surface, thereby inducing an osmotic force (i.e., depletion force) that results in aggregation of RBCs^26^,^27^. This force can be generated by both protein and non-protein polymers and explains the aggregating effects of large hydroxyethyl starch (>130 kDa) and Ficoll 400 kDa (F400; radius = 10 nm) which are commonly used to accelerate RBC sedimentation for leukocyte enrichment. Less appreciated, however, is that small polymers instead inhibit RBC aggregation^28^,^29^ by decreasing the osmotic force in the depletion layer owing to their ability to penetrate this confined space^27^,^30^. To understand whether this depletion model is consistent with our experimental findings with Ficoll polymers, we computed the interaction energies between RBCs in the presence of 70 kDa (radius = 5.1 nm) or 400 kDa Ficoll (radius = 10 nm) according to the theoretical formulation by Neu and Meiselman^26^. Here, we considered electrostatic repulsion forces due to the negatively charged glycocalyx on RBC surfaces as well as depletion forces induced by polymer exclusion, taking into consideration the spherical shape of Ficoll molecules in the calculation of osmotic properties. We found that Ficoll 70 kDa results in interaction energies that were universally non-negative, consistent with our experimental results. In contrast, the larger Ficoll 400 kDa leads to depletion layers thick enough (Fig. 2E) to reduce the total interaction energies to negative values (i.e., attractive forces) up to a concentration of ~14% (Fig. 2F). We further confirmed experimentally that 5% F400, but not 15%, led to RBC aggregation (Fig. 2G). These results extend the current literature on macromolecular depletion by demonstrating the applicability of the Neu and Meiselman model to Ficoll in addition to dextran and poly(ethylene glycol) (PEG) polymers which have been extensively studied. Further, comparison of RBC-aggregating properties of different polymers reinforces the notion that aggregation is determined by the hydrodynamic radii but not the molecular weights of polymers^27^. For instance, the highly branched dextrans occupy larger volumes in solution than Ficoll despite having similar molecular weights. As such, dextran 73 kDa (radius = 6.49 nm)^27^ but not Ficoll 70 kDa (radius = 5.1 nm) aggregates RBCs. Similarly, dextran 39.1 kDa (radius = 4.78 nm) also inhibits RBC aggregation^27^. These results have also been reproduced in our laboratory (data not shown).

In this study, we used the Stoke’s equation as a first approximation to understand how Ficoll prevented the settling of blood by modifying its properties (Fig. 2D). We found that the ability of F70 to inhibit RBC aggregation (i.e., preventing increases in particle radius *R*) was sufficient to explain its stabilization effects, while the alterations in viscosity and density were minimal and secondary. Dilution of WB with media (0% F70) did not stabilize blood as expected, likely because the dilution ratio was small and the associated decreases in RBC aggregation and viscosity—both resulting from the decreased plasma protein concentration—exerted opposing effects which led to insignificant net changes in settling. We note that this simple equation does not capture the time-dependent dynamics of aggregation; in practice, settling should accelerate as the radii of aggregates increase over time. Our calculation is therefore more representative of the 10% and 15% F70 conditions in which aggregation is absent—as observed in the agreement between experimental and estimated ESRs in these two F70 conditions (Figs 1B and 2D). The development of more complex models that incorporate the dynamics of aggregation is suitable for further research and is beyond the scope of the current study.

The interpretation of blood rheology (Fig. 2A,B) in WB and different F70 conditions requires a more detailed examination on the cellular (RBCs) and acellular (plasma) phases of blood. We first point out that WB exhibits shear-thinning behavior (Fig. 2A) because additional stress is required to disperse RBC aggregates at low shear rates (<50/s)^24^,^31^. The addition of F70 therefore leads to competing effects—it decreases blood viscosity by inhibiting RBC aggregation but at the same time increases the viscosity of plasma. The latter effect could be observed at the highest shear rates when RBC aggregates were completely dispersed (Fig. 2B; average viscosities were 4.1, 4.8, 7.9, and 11.6 mPa·s at 1000/s for WB, 5%, 10%, and 15% F70, respectively). At low shear rates, the decrease in viscosity in the cellular phase due to 5% and 10% F70 was comparable to the increase in the acellular phase, and therefore the viscosities did not change significantly. At 15% F70 however, there was no further inhibition in RBC aggregation, but instead the high polymer concentration led to a great increase in viscosity as well as shear-thinning behavior in the acellular phase.

Our approach of blood stabilization confers several benefits to the processing and cellular preservation of blood. First and foremost, the prevention of blood settling eliminates the need for continuous mixing, and may therefore facilitate storage and transportation as well as laboratory assays that require repeated, continuous sampling of blood. Red blood cells, which constitute 99% of the total volume of all blood cells, retained their biconcave morphology in Ficoll-stabilized blood. This result should benefit applications such as size-based cell sorting (e.g., filtration) or microfluidic processes that rely on hydrodynamic properties of cells such as deterministic lateral displacement^32^. Leukocytes, in particular the fragile neutrophils, displayed superior integrity and decreased apoptosis. Further, the inhibition of NET formation has important implications in blood storage. It is known that activated neutrophils release chromatin fibers mixed with neutrophil enzymes to form NETs^33^, which protect against infection but may also promote thrombosis^34^ and autoimmune reactions^35^. The release of NETs in stored RBC units for transfusion^21^ has thus been suggested to cause transfusion-related acute lung injury^36^. We hypothesize that the presence of NETs components—such as DNA, neutrophil elastase, and myeloperoxidase—induces collateral damage to other cells in WB and accelerates the degradation of the entire blood sample. As such, the preservation of all blood cells is critical even in applications where the cells of interest are non-hematologic. For instance, the analysis of rare circulating tumor cells (1 in 10^9^ blood cells) holds great potential as a non-invasive liquid biopsy for the clinical management of cancer^4^. Degradation of hematologic cells not only causes collateral damage to the rare tumor cells, but also negatively impacts cell sorting technologies that rely on defined biological and physical properties of blood cells.

An important property of Ficoll-stabilized blood is the compatibility with common cellular enrichment methods and downstream immunophenotyping assays. We found no impact on leukocyte enrichment using routine hypotonic lysis of RBCs, and the leukocytes could be further processed for neutrophil purification using a commercially available kit or subjected to flow cytometric analysis, which demonstrated no alterations in surface marker expressions. However, we note that in certain phenotyping applications—such as the PEG antiglobulin test—other polymers are introduced into the assay to enhance antibody binding. Whether the presence of F70 interferes with the intended functions of these polymers warrants further investigation.

The easy implementation of our protocol and availability of Ficoll polymers may provide an opportunity to improve existing procedures. For instance, immunological assays are sometimes performed with a 24-hour delay to account for specimen shipping. Short-term storage of cord blood for up to 48 hours prior to cell enrichment and cryopreservation is also a common practice. Within these time frames, the degrading granulocytes (in which >90% are neutrophils) have been found to negatively impact T-cell assays^37^,^38^. The ability of Ficoll in maintaining cellular integrity especially within these shorter time frames (16.3 ± 6.8% Sytox-positive in WB vs. 8.0 ± 1.9% in 10% F70, *p* < 0.05 at 48 hours) may find immediate use in similar applications.

## Conclusions

This work demonstrates a simple yet effective biophysical strategy for the preservation of whole blood samples. By exploiting the ability of small polymers in preventing RBC aggregation, we succeeded in stabilizing blood in a homogeneous suspension, improving the preservation of blood cells, and limiting the release of cytotoxic breakdown products. These biological benefits combined with practical advantages in the storage and handling of stabilized blood samples should be of value in medical diagnostics, transfusion medicine, and blood-on-a-chip type devices. The concept of physical stabilization of whole blood is a relatively unexplored topic, and our study lays the foundation for further improvement through the integration of biochemical preservation strategies.

## Methods

### Blood samples and addition of Ficoll

Blood samples with normal hematologic parameters were obtained from healthy donors in Massachusetts General Hospital (MGH) or purchased from Research Blood Components (Brighton, MA). Informed consent was obtained from all donors at MGH and all experiments were approved by the MGH Institutional Review Board; all research methods were carried out in accordance with the approved guidelines. Blood samples were drawn into Acid Citrate Dextrose-A tubes (BD Vacutainer; 8.5 mL) and used within four hours.

Whole blood was used without any modification. To introduce F70 (GE Healthcare) polymers into blood, concentrated stock solutions of F70 (20%, 40%, and 60% w/v) were dissolved into RPMI 1640 Medium (without phenol red; Life Technologies) supplemented with 10 mM HEPES (Life Technologies), filter-sterilized, and added to WB at a volumetric ratio of 1:3. For example, to prepare blood with 5% F70, 1 part 20% F70 was added to 3 parts WB and mixed by inversion (HulaMixer; Life Technologies) for 10~15 minutes before use. For 0% F70 samples, 1 part RPMI without F70 was added to 3 parts WB. Therefore all Ficoll-blood samples were consistently diluted to a 75% blood volume fraction; we did not standardize dilution according to hematocrit. We also note that the contribution of F70 to the osmolarity of blood is minimal (15% F70 corresponds to an increase of 2.14 mM which results in <1% change from the physiologic 300 mM). Blood samples were stored in sterile, air-tight tubes undisturbed at room temperature.

### Erythrocyte settling rate assay

The ESR assay was performed with 1.3 mL of WB or Ficoll-blood using pipets that conform to the dimensions of the standardized Westergren method (Dispette 2; Fisherbrand). The ESRs in millimeters were recorded at 24, 48, and 72 hours.

### Rheology and density measurement

To ensure accurate viscosity measurements especially at low shear rates, Couette rheometry was performed using a TA Instruments Discovery HR-3 rheometer with a steel double-wall concentric cylinder geometry, which holds 8.5 mL of sample with an operating gap of 2000 μm. Data points were acquired at shear rates ranging from 0.1/s to 1000/s at 5 points per decade. The equilibrium criterion was to obtain three consecutive torque values that were within 5% of each other at each imposed shear rate; such conditions ensure that data acquisition takes place when RBC aggregation and their dispersal by the imposed shear reach an equilibrium state. Densities of WB or Ficoll-blood samples were obtained by measuring the weight increase of a pipet tip after pipetting up exactly 1 mL of sample.

### Estimation of erythrocyte settling rate

The ESR was estimated based on the settling velocity 
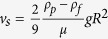
 derived from the settling of a single particle in a Newtonian fluid, where *ρ*_*p*_ is the sphere density, *ρ*_*f*_ is the fluid density, *μ* is the fluid viscosity, *g* is gravity, and *R* is the radius of the sphere. Here, the RBC was assumed to be a sphere with a radius of 4 μm and density of 1.1 g/cm^3^. Fluid density and viscosity used the measured values (at the lowest shear rate of 0.1/s in the case of viscosity) of the blood or Ficoll-blood samples. This method effectively treats the entire sample (both cellular and acellular components) as a homogenous fluid.

### Morphological assessment of blood cells

For the enumeration of echinocytes, samples were gently mixed before a drop (~10 μL) was transferred to a glass slide, smeared, and imaged immediately using phase-contrast microscopy at 40 × using an EVOS FL Cell Imaging System (Life Technologies). About 100 random RBCs were counted per sample and echinocytes were identified by their distinct spiculations.

Wright-Giemsa staining was performed according to standard procedures. Briefly, a drop (~10 μL) of sample was smeared on a glass slide, air-dried, fixed in 100% methanol, and dipped in Wright-Giemsa stain (Sigma) for 30 seconds before rinsing in deionized water. Images were captured with a Nikon DS-Ri1 color camera (12-bit; 1280 × 1024 resolution) using a Nikon 100 × Apo VC 100 × /1.40 oil objective on a Nikon Eclipse 90i microscope.

### Leukocyte enrichment and yield quantification

Red blood cell lysis was performed using Red Blood Cell Lysis Solution (Miltenyi Biotec). After lysis, cells were spun down at 300 × g, washed with 10 mL RoboSep buffer (Miltenyi Biotec), spun again, resuspended in 600 μL RoboSep buffer, and counted using a Beckman Z2 Coulter Counter. Neutrophil enrichment was performed using the EasySep Human Neutrophil Enrichment Kit (Stemcell Technologies) according to manufacturer’s protocol. Briefly, the depletion antibody cocktail was mixed with the enriched leukocytes obtained by RBC lysis followed by incubation with magnetic particles. The EasySep Magnet was then used to immobilize unwanted cells as the label-free neutrophils were poured into another conical tube. Enriched neutrophils were then re-spun and resuspended in 1 mL RPMI media containing 0.3% BSA and 10 mM HEPES, counted, and stained for imaging flow cytometry.

### Imaging flow cytometry for surface markers and cell viability

Imaging flow cytometry was performed using the ImageStream^X^ Mark II imaging flow cytometer (Amnis Corporation) equipped with a 40 × objective, 6 imaging channels, and 405 nm, 488 nm, and 642 lasers. For analysis of cell viability and CD45 expression, the enriched leukocytes were resuspended in 0.1% BSA in HEPES-buffered saline after RBC lysis and stained with the following antibodies and stains where applicable: DRAQ5 (1 μM; Cell Signaling Technologies), Sytox Blue (1 μM; Life Technologies), CellEvent Caspase-3/7 Green Detection Reagent (0.75 μM; Life Technologies), FITC-conjugated CD45 antibody (1:500; clone 5B1; Miltenyi Biotec), PE-conjugated CD66b antibody (1:125; clone G10F5; Stemcell Technologies), and PE-Cy7-conjugated CD16 antibody (1:200 or 1:333; clone 3G8; BD Biosciences). Single cells were gated using the nuclear marker DRAQ5. Neutrophils were identified by the dual positivity of CD66b and CD16. For analysis of neutrophil activation post-enrichment, cells were stained with DRAQ5 (1 μM; Cell Signaling Technologies), VioBlue-conjugated CD45 antibody (1:100; clone 5B1; Miltenyi Biotec), Alexa Fluor 488-conjugated CD11b antibody (1:500; clone ICRF44; Stemcell Technologies), PE-conjugated CD66b antibody (1:125; clone G10F5; Stemcell Technologies), and PE-Cy7-conjugated CD16 antibody (1:333; clone 3G8; BD Biosciences).

### Immunofluorescence staining and microscopy for visualization of NETs

Blood smears on poly-L-lysine-coated glass slides were fixed in 100% methanol, air-dried, fixed with 4% paraformaldehyde, and blocked and permeabilized (2% goat serum + 0.1% Triton X-100) for 4 hours at room temperature. Slides were then incubated with anti-neutrophil elastase rabbit pAb (25 μg/mL; Calbiochem) and anti-H2A-H2B-DNA mouse mAb (clone PL2-6; 1 μg/mL ; gift of Marc Monestier, Temple University, Philadelphia, PA) in 0.3% bovine serum albumin overnight at 4 °C. Next, the slides were incubated with Alexa Fluor 488-conjugated goat anti-rabbit IgG and Alexa Fluor 555-conjugated goat anti-mouse IgG (both 1:500; Life Technologies) for 45 minutes at room temperature, rinsed with PBS, and mounted using VECTASHIELD Mounting Medium with DAPI (Vector Laboratories). Images were captured with a QImaging Retiga 2000R camera using a Nikon S Plan Fluor ELWD 60 × /0.70 objective on a Nikon Eclipse 90i microscope.

### Quantification of neutrophil elastase

Blood samples were mixed gently, warmed to 37 °C for four hours for the release of NETs contents into the plasma, and diluted with PBS to a final blood volume fraction of 25% before centrifugation at 2000 × g for 5 minutes. The supernatant was then carefully transferred to a new centrifuge tube and stored at −80 °C for further processing. The level of neutrophil elastase was quantified using the Neutrophil Elastase Activity Assay Kit (Cayman Chemical Company) according to manufacturer’s protocol, using a SpectraMax M5 spectrometer (Molecular Devices). As a positive control for NETosis, phorbol myristate acetate (100 nM) was added to fresh WB prior to incubation.

### Statistical analyses

Numerical data are reported as mean ± standard deviation. Pairwise comparisons used the Mann-Whitney test. For comparisons of densities, we used 1-way ANOVA followed by the posttest for linear trend. For comparisons of WB and F70 conditions over time, we used 2-way ANOVA followed by the Bonferonni posttest for pairwise comparisons. All statistical analysis was performed with Prism 5 (GraphPad).

## Additional Information

**How to cite this article**: Wong, K. H. K. *et al.* The Role of Physical Stabilization in Whole Blood Preservation. *Sci. Rep.* **6**, 21023; doi: 10.1038/srep21023 (2016).

## Supplementary Material

Supplementary Information
