# Supplementary material for: The Role of Physical Stabilization in Whole Blood Preservation
Source: Sci Rep. 2016 Feb 15;6:21023. doi: 10.1038/srep21023 (PMC4753451; doi:10.1038/srep21023)

The Role of Physical Stabilization in Whole Blood Preservation

Keith H. K. Wong^1^, Rebecca D. Sandlin^1^, Thomas R. Carey^1^, Kathleen L. Miller^1^, Aaron T. Shank^1^, Rahmi Oklu^2,3^, Shyamala Maheswaran^4^, Daniel A. Haber^5,6^, Daniel Irimia^1^, Shannon L. Stott^7^ and Mehmet Toner*^1^

^1^BioMEMS Resource Center, Center for Engineering in Medicine, & Department of Surgery, Massachusetts General Hospital, Harvard Medical School, Boston, MA 02114

^2^Department of Radiology, Massachusetts General Hospital, Harvard Medical School, Boston, MA 02114

^3^Mayo Clinic, Division of Interventional Radiology, Scottsdale, AZ 85259

^4^Cancer Center & Department of Surgery, Massachusetts General Hospital, Harvard Medical School, Boston, MA 02114

^5^Cancer Center & Department of Medicine, Massachusetts General Hospital, Harvard Medical School, Boston, MA 02114

^6^Howard Hughes Medical Institute, Chevy Chase, MD 20815

^7^Cancer Center, Department of Medicine, & BioMEMS Resource Center, Center for Engineering in Medicine, Massachusetts General Hospital, Harvard Medical School, Boston, MA 02114

*Corresponding author

E-mail: [mtoner@hms.harvard.edu](mailto:mtoner@hms.harvard.edu)

Telephone: 617 724 5336

Fax: 617 724 2999

**Supplementary Methods**

We investigated whether the removal of F70 post-storage would reverse its protective effects on RBCs. To wash the blood sample extensively, we diluted it 10 times with PBS, centrifuged it at 200×g for 3 minutes, removed the supernatant, and repeated this procedure one more time before resuspending the sample in 100 times its original volume. The final concentration of F70 was therefore diluted at least 10000 times. We then enumerated echinocytes using phase-contrast images obtained at 40× using an EVOS FL Cell Imaging System (Life Technologies).

**Supplementary Figure S1.**

Percentages of echinocytes after storage in WB or 10% F70 for 72 hours, quantified after extensive washing (*p* = 0.0175, Mann-Whitney test).

**Supplementary Figure S1.**


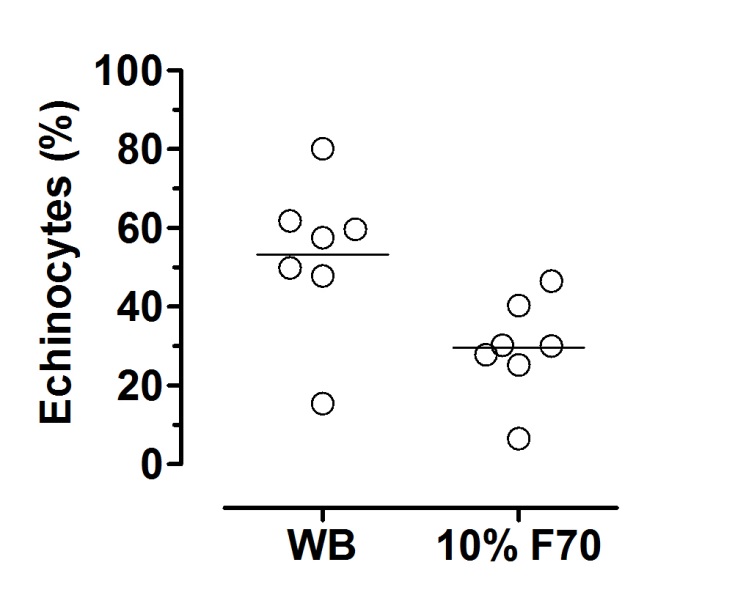

Supplement: Supplementary Information [file srep21023-s1.doc]
